# The difference between standing and sitting in 3 different seat inclinations on abdominal muscle activity and chest and abdominal expansion in woodwind and brass musicians

**DOI:** 10.3389/fpsyg.2014.00913

**Published:** 2014-08-25

**Authors:** Bronwen J. Ackermann, Nicholas O'Dwyer, Mark Halaki

**Affiliations:** ^1^School of Medical Sciences, Sydney Medical School, The University of SydneySydney, NSW, Australia; ^2^School of Human Movement Studies, Charles Sturt UniversityBathurst, NSW, Australia; ^3^Discipline of Exercise and Sport Science, The University of SydneySydney, NSW, Australia

**Keywords:** wind musicians, surface electromyography, respiratory inductive plethysmography, posture, lung function

## Abstract

Wind instrumentalists require a sophisticated functioning of their respiratory system to control their air stream, which provides the power for optimal musical performance. The air supply must be delivered into the instrument in a steady and controlled manner and with enough power by the action of the expiratory musculature to produce the desired level of sound at the correct pitch. It is suggested that playing posture may have an impact on the abdominal muscle activity controlling this expired air, but there is no research on musicians to support this theory. This study evaluated chest and abdominal expansion, via respiratory inductive plethysmography, as well as activation patterns of lower and upper abdominal musculature, using surface electromyography, during performance of a range of typical orchestral repertoire by 113 woodwind and brass players. Each of the five orchestral excerpts was played in one of four randomly allocated postures: standing; sitting flat; sitting inclined forwards; and sitting inclined backwards. Musicians showed a clear preference for playing in standing rather than sitting. In standing, the chest expansion range and maximum values were greater (*p* < 0.01), while the abdominal expansion was less than in all sitting postures (*p* < 0.01). Chest expansion patterns did not vary between the three sitting postures, while abdominal expansion was reduced in the forward inclined posture compared to the other sitting postures (*p* < 0.05). There was no significant variation in abdominal muscle activation between the sitting postures, but the level of activation in sitting was only 2/3 of the significantly higher level observed in standing (*p* < 0.01). This study has demonstrated significant differences in respiratory mechanics between sitting and standing postures in wind musicians during playing of typical orchestral repertoire. Further research is needed to clarify the complex respiratory mechanisms supporting musical performance.

## Introduction

Playing a wind instrument is one of the most strenuous activities of the respiratory system (Deniz et al., 2006), with the requirement to blow into resistances of up to 120 mmHg creating extraordinary ventilatory demands (Fuks and Fadle, 2002). Wind instrumentalists also require a sophisticated functioning of their respiratory system to control all aspects of their air stream for optimal musical performance (Gilbert, 1998). To produce a note, a column of air must pass through a vibrator mechanism—the lips or a single or double reed system—into a brass or woodwind instrument in a steady and controlled manner, and with enough power to produce the desired level of sound at the correct pitch (Sataloff et al., 1998). The air provides the power behind all wind playing, while the reed apparatus or lip opening controls much of the air column vibration and output rate (Farkas, 1962).

The ventilatory demands of playing a wind instrument change according to musical requirements and require changes in the intra-oral and intra-thoracic pressure, mouthpiece pressure and flow rates (Iltis, 2003). Hence the control of the flow of air is critical and this directly impacts respiratory muscle function (Webster, 2014). It is likely that a number of different muscle recruitment strategies are necessary to achieve the complexity of breathing patterns involved in producing sound on a wind instrument (Sataloff et al., 1998). However, while many wind music pedagogues advocate correct breathing as being essential for musical performance, little evidence or consensus advice exists as to how this should be done (Sehmann, 2000).

Lung function tests in musicians have produced conflicting results over the years. While some authors have not found a significant difference in basic spirometry measures between wind musicians and controls (Schorr-Lesnick et al., 1985; Fiz et al., 1993; Fuhrmann et al., 2011), others have found reduced (Akgun and Ozgonul, 1967; Deniz et al., 2006) or increased lung function (Bouhuys, 1964; Zuskin et al., 2009; Khuje and Hulke, 2011). Given these conflicting findings, further study of spirometry measures in wind musicians appears warranted.

It has been suggested previously that respiratory function of wind musicians may be directly affected by their playing posture (Brandfonbrener and Kjelland, 2002; Webster, 2014). This is an important consideration given the wide variety of chairs that musicians may encounter in different playing venues. They may also be required to play while standing. However, no evidence exists regarding the impact of playing posture on respiratory muscle activity in wind and brass musicians.

In other contexts, different postural conditions have been shown to affect patterns of muscle activation and measures of lung function. Standing appears to increase activation of the oblique abdominal muscles, perhaps related in part to increased pressure on the abdominal wall by the abdominal contents (De Troyer, 1983; Kera and Maruyama, 2005). In normal individuals sitting in a slouched position, respiratory effort is increased compared to normal sitting, while there is a simultaneous decrease in respiratory capacity and breathing control (O'Sullivan et al., 2002; Landers et al., 2003; Lin et al., 2006). Furthermore, using respiratory inductive plethysmography (RIP), the expansion characteristics of the thoracic and abdominal cavity have been shown to vary according to different postures (Lee et al., 2010). This tool has proved useful in previous studies of wind musicians (Cugell, 1986; Fuks and Sandberg, 1999), providing valuable information on their respiratory mechanics during performance, although more research is needed in this area.

The aim of this study was to investigate respiratory movements and abdominal muscle activity under four different postural conditions while playing a range of classical music excerpts on a woodwind or brass instrument. It was hypothesized that abdominal and chest expansion, and abdominal muscle activity, would change significantly between standing and sitting postures; and that abdominal muscle activity would vary significantly between different sitting postures, with muscle activity lowest in the backward inclined posture.

## Methods

### Selection and description of participants (demographics-age, sex, instrument)

An invitation to participate in this study was extended to orchestral musicians. Of the 113 woodwind and brass players who volunteered (68 males and 45 females), there were 74 professionals and 39 students with a mean age (±standard deviation) of 34.1 (±12.7) years, as detailed in Table 1.

**Table 1:** **Musician numbers for each instrument and musical excerpts played**.

**Instrument**	**Number**	**Professional**	**Males**	**Max. Pressure (mmHg) (13)**	**Excerpt 1**	**Excerpt 2**	**Excerpt 3**	**Excerpt 4**	**Excerpt 5**
Oboe	11	10	6	80.8	Ravel, Bolero	Smetena, Die verkaufte Braut	Beethoven, Fidelio	Bartok, Konzert fur Orchestra	Tchaikovsky, Symphony 4
Cor anglais	3	3	–	54.7	Ravel, Bolero	Smetena, Die verkaufte Braut	Beethoven, Fidelio	Bartok, Konzert fur Orchestra	Tchaikovsky, Symphony 4
Clarinet	12	8	6	86.4	Mendellsohn, Saltarello	Rossini, Semeramis	Tchaikovsky, Symphony 6	Tchaikovsky, Franscesca De Rimini	Tchaikovsky, Symphony 5
Bass clarinet	2	2	2	86.4^*^	Aida	Puccini, Madame Buuterfly	Tchaikovsky, Nutcracker	Tchaikovsky, Franscesca De Rimini	Tchaikovsky, Symphony 5
Flute	23	10	11	77.8	Bolero, Ravel	Beethoven, Leonore Overture	Prokofiev, Peter and the Wolf	Mendelssohn, Midsummer night's dream	Ravel, Daphnis and Chloe
Bassoon	11	7	6	89.7	Tchaikovsky, Nutcracker Suite	Tchaikovsky, Symphony 6	Rimsky-Korsakov, Scherezade	Mussorgsky	Stravinsky, Rite of Spring
Contra bassoon	3	3	1	89.7^*^	Mahler, Symphony 9	Ravel, Concerto for the left hand	Ravel, Mother Goose	Brahms, Sinfonie in C	Beethoven 5
French horn	10	9	5	115.9	Wagner, Siegfried	Beethoven, Symphony 7	Mahler, Symphony 1	Mussorgsky, Pictures at an Exhibition	Strauss, Till Eulenspiegel
Trombone	9	6	9	126	Ravel, Bolero,	Wagner, Die Walküre	Wagner, Die Walküre	Straus, Till Eulenspiegel	Berlioz, Hungarian March
Bass trombone	2	2	2	126^*^	Beethoven 9	Wagner, Valkeries	Brahms Symphony 1	Haydn—The Creation	Berlioz, Hungarian March
Trumpet	16	7	11	125.8	Wagner, Parsifal Prelude	Mahler Symphony 5	Bizet, Carmen	Rimsky Korsakoff: Scheherazade	Stravinsky, Petrushka
Tuba	4	1	4	77.6	Mahler, Symphony 1	Wagner, Die Walküre	Wagner, Meistersinger	Strauss, Ein Heldenleben	Berlioz, Hungarian March
Saxophone	3	2	1	56.2	Glazounov's Concerto for saxophone in Eb	Bizet, Larlesienne 2nd movement, intermezzo	Gershwin Rhapsody in Blue	Honegger—Mouvement Symphonique No. 3	Khachaturian, Saber Dance
Recorder	1	1	1	11.6	Johan Jacob van Eyck, Amarilli mia bella	Brandenburg Concerto No. 2	Hirose, Meditation	Anton Heberle, Sonate brilliante for Csakan or recorder, 2nd movement	Telemann, Duet for Flute and violin, 3rd movement
Shakahatchi	1	1	1	77.8^*^	Himeru no Omoi	Shingetsu	Daha	Takiochi	Reibo
Piccolo	2	2	2	77.8^*^	Bolero, Ravel	Beethoven, Leonore Overture	Tchaikovsky, Symphony 4	Mendelssohn, Midsummer night's dream	Ravel, Daphnis and Chloe

* *Estimated values*.

### Ethics

This project was approved by the University of Sydney Human Ethics Committee (protocol number 2012/1255).

### Equipment

Spirometry (EasyOne, ndd Medical Technologies, Inc, Andover, MA, USA) was used to measure each participant's ventilator capacity. The following measures were obtained: Forced Vital Capacity (FVC), Peak Expiratory Flow (PEF), Forced Expiratory Volume in 1 second (FEV1) and the proportion of the total lung volume that can be expired in 1 second (FEV1/FVC).

The activation of the selected abdominal muscles was measured using electromyography (EMG). The skin was prepared with an abrasive gel (Nuprep, DO Weaver and Co., Aurora, CO, USA) and alcohol. To measure abdominal activity, placement for internal and external oblique musculature was used as described by Kera and Maruyama (2005), with pairs of silver/silver chloride surface electrodes (Red Dot, 2258, 3M, London, Ontario, Canada) were placed ~2.5 cm apart, and a ground electrode was placed over the iliac crest. The authors of the current paper felt that the placement could not be considered to exclusively represent these muscles, and so chose instead to refer to these as “upper” (external oblique) and “lower” (internal oblique) abdominal muscles. Prior to application, the musicians were asked to perform a forward trunk flexion task to activate rectus abdominis in order to place electrodes lateral to this muscle. This muscle had been previously found to not be sensitive to postural change in relation to respiration by Kera and Maruyama. EMG signals were amplified (gain=1000) and band-pass (10–500 Hz) filtered (EMG 100B, Biopac Systems Inc, Goleta, CA, USA).

Respiratory inductance plethysmography (Calibrator Inductotrace System, Ambulatory Monitoring Inc., Ardsley, NY, USA) was used to measure the expansion of abdominal and chest cavities. A microphone (AKG C420, AKG Acoustics GmbH, Vienna, Austria) was used to synchronize the EMG signals, abdominal and chest movement with the music. All signals were sampled at a rate of 2000 Hz using a 16-bit A/D converter (MP100A, Biopac Systems) and AcqKnowledge software (v.3.9.0, Biopac Systems).

### Procedures

Maximum voluntary isometric contractions (MVCs) designed to produce maximal activity in the external and internal obliques (based on Duiverman et al., 2004) were conducted and the EMG signals recorded. Each MVC was repeated three times with at least a 30-s break between trials. These MVCs were conducted in standing, and chest expansion maximums were conducted in both sitting and standing.

With the plethysmography bands in place around the abdominal and chest cavities, as previously described in Lee et al. (2010), the participants' ventilatory capacity was measured. During this procedure, the participants were instructed to breathe in maximally and once the spirometer was in place, quickly and fully exhale. This procedure was repeated at least three times until the spirometer indicated the test was complete.

**Figure 1:**
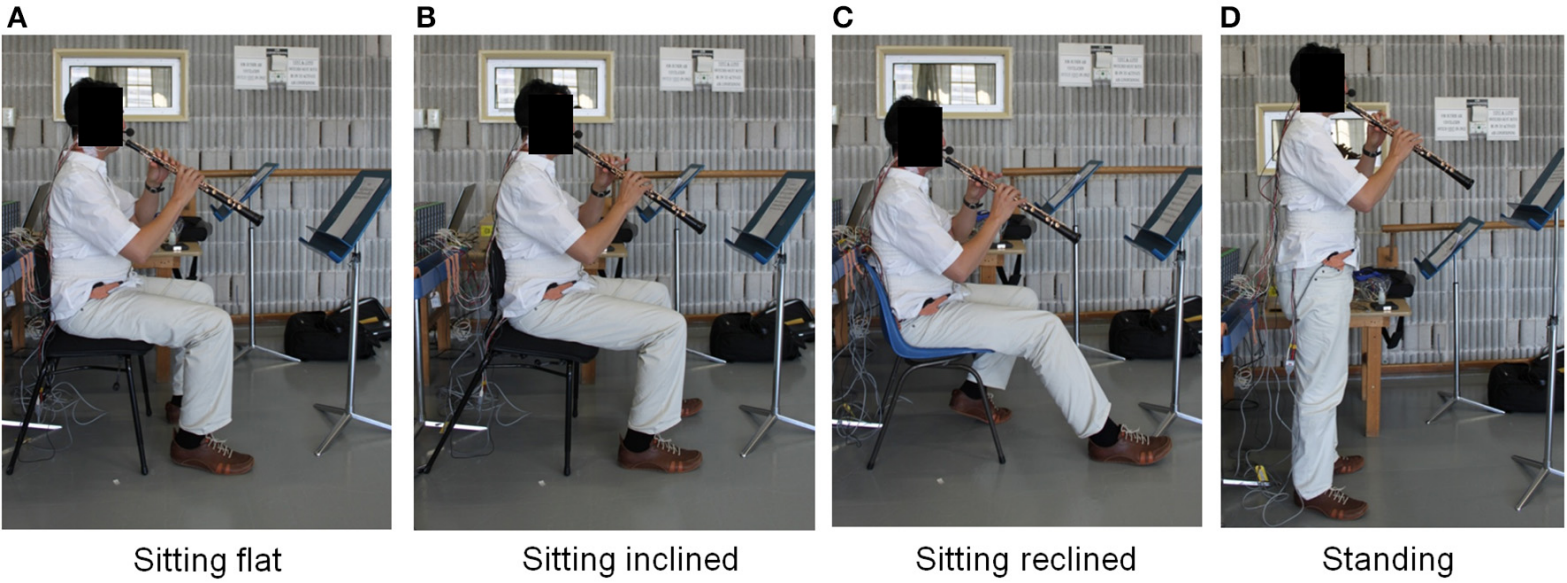
**The four different playing postures: (A) sitting flat, (B) sitting inclined down and forward 10°, (C) sitting reclined down and backward 10°, (D) standing**.

Participants were then asked to play five different musical excerpts (Table 1) in the four different postures illustrated in Figure 1. The postures were standing, sitting with the seat flat, sitting with the seat inclined 10° down and forward and sitting with the seat reclined 10° down and backward. The standing posture was included because most wind musicians practice in standing and this is also the posture adopted for solo performance. As well as a typical flat seat base position, the slouch sitting or 10° reclined posture was chosen based on the slope of typical commercial chairs found in many music schools, and also because of the evidence from other populations suggesting that this posture would impair breathing performance (O'Sullivan et al., 2002; Kera and Maruyama, 2005). The 10° forward tilt sitting posture was included because it has been suggested previously to enhance breathing function in musicians (Norris, 1993). In the given chair adjusted condition, musicians were asked to sit as if performing as normally as possible. This was done in an attempt to create as realistic an indication of respiratory characteristic without adding additional constraints or variations to their performance actions.

Most of the participants were already familiar with the musical excerpts, but they were given time to briefly practice the excerpts prior to testing. To improve the consistency of the breathing across each performance, a metronome and breathing markings were placed on the musical scores. The ordering of the excerpts and postural conditions was randomized. The musicians also rated the postures from most to least favorite.

### Signal processing

Matlab (v. 2010, The Mathworks, Natick, MA, USA) was used for all signal processing. The EMG signals were high-pass filtered (10 Hz, Butterworth, zero-lag, 8th-order), rectified and low-pass filtered (10 Hz, Butterworth, zero-lag, 8th-order). The EMG signals recorded during each of the musical excerpts and postures were normalized to the maximum signals recorded during the MVCs and expressed as % MVC.

The abdominal and chest expansion signals were normalized to the maximum and minimum expansion values recorded during the ventilatory capacity test and reported as % total volume. In order to compensate for any signal drift in the plethysmography system, the expansion level at the beginning of each breath was subtracted first, using the following equation:

$$\frac{\text{expansion }-\text{ expansion at start of each breath}}{\text{maximum expansion }-\text{ minimum expansion}}\;\times \;100\%$$

**Figure 2:**
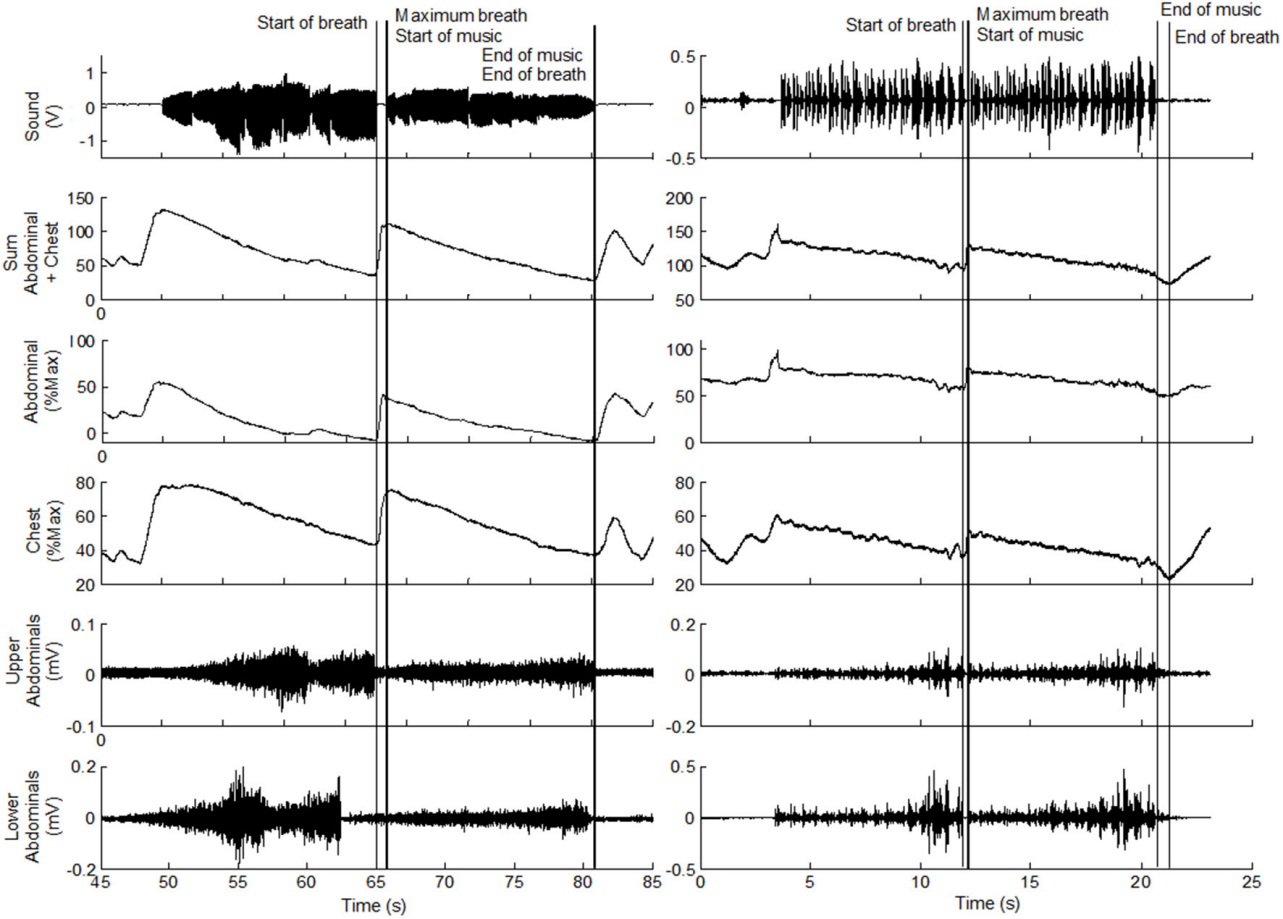
**Samples of the sound intensity, abdominal and chest expansion and upper and lower abdominal EMG signals for a trumpet player (left) and an oboe player (right) during sitting flat**. The Start of breath, Maximum breath, Start of music, End of music and End of breath are indicated for one breath cycle.

Five distinct event time points were identified for each breath taken during the performance, as shown in Figure 2, and were defined as:

Start of breath: the minimum value of the sum of the abdominal and chest expansion signals just before the audio signal was detected.

Maximum breath: the maximum value of the sum of the abdominal and chest expansion signals just before the audio signal was detected.

Start of music: the point at which any audio signal was detected.

End of music: the point at which the audio signal ceases.

End of breath: the minimum value of the sum of the abdominal and chest expansion signals just after or when the audio signal ceased.

### Statistical analysis

Normality of the data was checked and confirmed using probability plots. Factorial analyses of covariance (ANCOVAs) (within subject factors: posture and event time points, covariate: maximum pressure) were used to investigate differences in the average activity levels of abdominal muscles and the average expansions of the abdomen and chest across excerpts (Statistica, Version 10, StatSoft, Inc., USA). The number of levels for the event time points were 5 for the muscle activity but were 4 for the abdominal and chest expansion, because expansion was always zero at the start of breathing, so only four time points were used (see Figure 2). ANCOVAs (within subject factors: posture, covariate: max pressure) were used to investigate differences in the range of abdominal and chest expansion. Similar instruments were grouped together to form the following 10 musician groups: flute/sakahatchi/piccolo (*N* = 26), oboe/cor anglais (*N* = 14), bassoon/contra bassoon (*N* = 14), clarinet/bass clarinet (*N* = 14), French horn (*N* = 10), trombone/bass trombone (*N* = 11), trumpet (*N* = 16), tuba (*N* = 4), saxophone (*N* = 3), recorder (*N* = 1). Single factor ANOVAs were used to test differences between musician groups for each of the lung function variables. Bonferroni *post-hoc* test was used when significant ANOVA results were obtained. A Chi-squared test for goodness of fit was used to assess whether some positions were preferred over others. The level of significance was set at *p* < 0.05.

## Results

**Figure 3:**
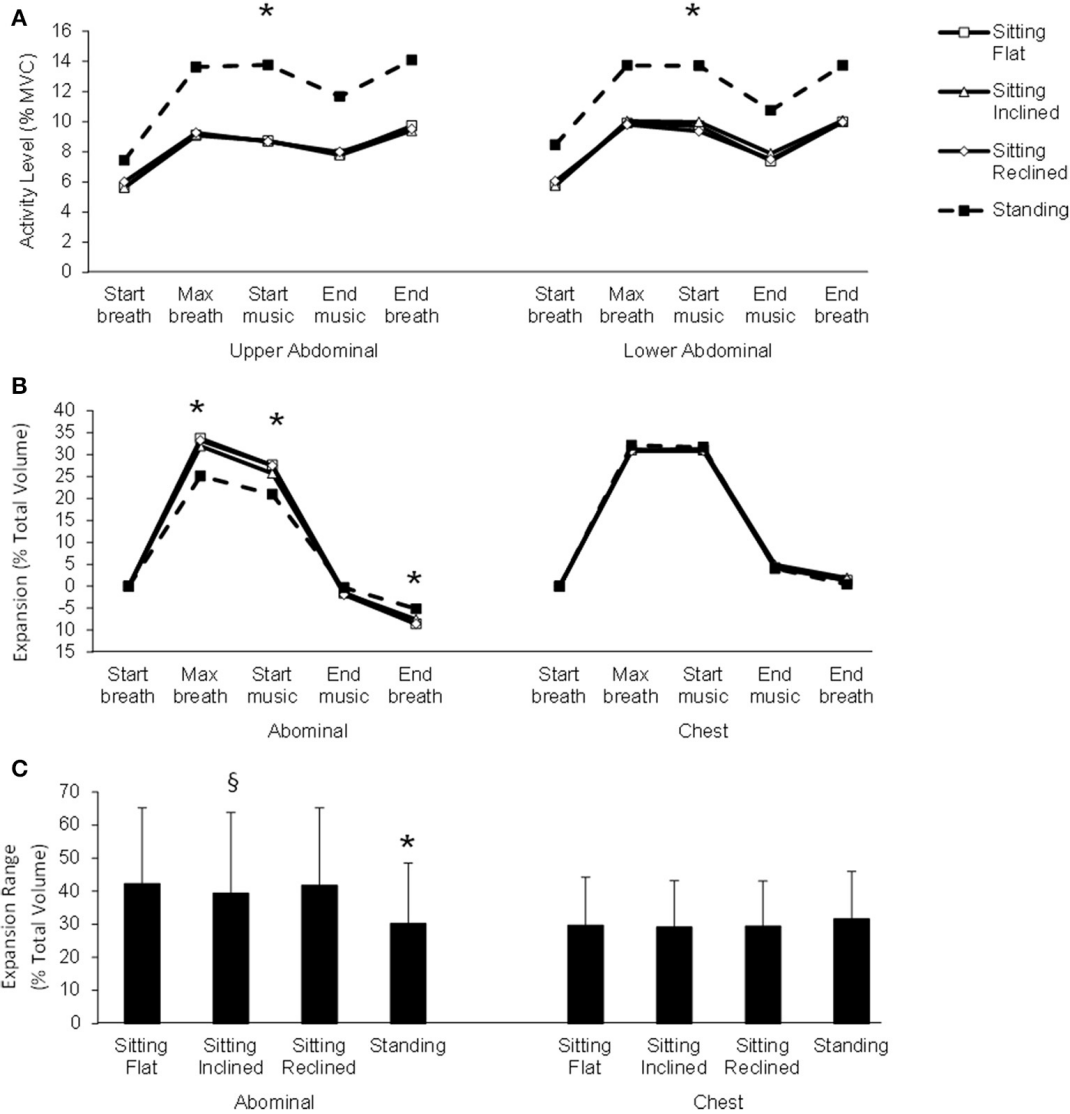
**(A)** Mean activity levels in the upper and lower abdominal muscles and **(B)** mean abdominal and chest expansion for the four postures at the five time points. **(C)** Mean ± standard deviation of the range (maximum—minimum) of abdominal and chest expansions for the four postures. ^*^ indicates significant difference between standing and all sitting postures and ^§^ indicates significant difference between sitting inclined and sitting flat.

The mean activity levels in the upper and lower abdominal muscles are shown for the four different postures at the five event time points in the breath cycle in Figure 3A. The activity levels varied significantly between the postures [*F*_(3, 333)_ ≥ 20.2, *p* < 0.02], with *post-hoc* tests showing no significant difference among the three sitting postures with mean activity levels of approximately 8% MVC in both muscles (*p* = 1.00), but higher activity during standing where mean activity increased to approximately 12% MVC in both muscles (*p* < 0.001). The activity levels changed with time across the breath cycle [*F*_(4, 444)_ ≥ 13.4, *p* < 0.04], with the least activity at the start (*p* < 0.001), an increase at the maximum breath (*p* < 0.001) which was unchanged at the start of the music (*p* = 1.00), a decrease in activity at the end of the music (*p* ≤ 0.05), and finally an increase at the end of the breath (*p* < 0.001). There were no significant interactions among any of the postures, time and/or max pressure (*p* ≥ 0.19).

The chest cavity expansion (Figure 3B) was significantly different across the time points [*F*_(3, 333)_ = 37.14, *p* < 0.001] but was not different among the postures [*F*_(3, 333)_ = 1.31, *p* = 0.27]. There were no significant interactions among any of the postures, time and/or max pressure (*p* ≥ 0.24). The range of change in the chest cavity expansion (Figure 3C) was not different between postures [*F*_(3, 333)_ = 0.17, *p* = 0.91].

The abdominal cavity expansion was significantly different across the time points [*F*_(3, 333)_ = 26.30, *p* < 0.001]. The variation of abdominal expansion across the time events was different for the different postures [*F*_(9, 999)_ = 4.14, *p* < 0.001]. The *post-hoc* tests showed that the abdominal expansion in standing was lower at maximum breath (by ~9% of total volume) and start of music (by ~7% of total volume) (*p* < 0.001) and higher at the end of the breath (by ~1% of total volume) (*p* < 0.04) than all the sitting postures, with no significant differences among the sitting postures (*p* ≥ 0.76). There were no other significant differences found between musician groups (*p* > 0.07). The range of change in the abdominal cavity expansion was different between postures [*F*_(3, 333)_ = 4.82, *p* < 0.003]. *Post-hoc* tests showed that there was a lower range in standing (30.3 ± 18.3 % total volume) than all the sitting postures (41.2 ± 23.5 % total volume) (*p* < 0.001) and in sitting inclined (39.5 ± 24.3 % total volume) compared to sitting flat (42.3 ± 23.0 % total volume) (*p* < 0.04). No other differences were found among sitting postures (*p* ≥ 0.12).

The lung function tests revealed that the musicians had normal lung function with mean ± standard deviation values very close to predicted values for FVC (102 ± 12%), FEV1 (98 ± 14%), FEV1/FVC (99 ± 10%) and PEF (98 ± 19%). There were no significant other differences in any of these variables between the musician groups [*F*_(9, 102)_ ≤ 1.42, *p* ≥ 0.19].

Standing was rated most often as the favorite playing posture, followed by sitting flat, then sitting inclined, with sitting reclined most frequently being the least favorite (Table 2). The χ^2^ was not significant (*p* = 0.37) for sitting inclined indicating that proportion of musicians who ranked it 1st, 2nd, 3rd, and 4th were similar. Sitting flat was the most frequent second and third favorite posture and showed the lowest score from musicians rating it as the least favorite.

**Table 2:** Musicians' ratings^*^ of the four postures.

	**Sitting Flat χ^2^_(3, *N* = 84)_ = 30.7, *p* < 0.001**	**Sitting Inclined χ^2^_(3, *N* = 84)_ = 3.14, *p* = 0.37**	**Sitting Reclined χ^2^_(3, *N* = 84)_ = 85.6, *p* < 0.001**	**Standing χ^2^_(3, *N* = 84)_ = 77.6, *p* < 0.001**
Favorite posture	21%	18%	1%	65%
Second favorite	43%	27%	10%	20%
Third favorite	33%	31%	23%	10%
Least favorite	2%	24%	67%	5%

* *Percentage of musicians who ranked each posture in each category*.

## Discussion

In this first study providing in-depth data on the impact of posture on breathing mechanics during musical performance in wind and brass instruments, a clear preference was found for playing in a standing posture rather than sitting. This preference was associated with markedly increased activation of abdominal muscles, clearly decreased magnitude and range of expansion of the abdomen, and slightly increased magnitude and range of expansion of the chest (Figure 3). This result confirms the previous finding that activation of abdominal musculature is increased in the standing posture (De Troyer, 1983; Kera and Maruyama, 2005). De Troyer (1983) suggested that this increase may be related to the effect of gravity on the abdominal contents. In contrast, there were no significant differences in muscle activation between the three different sitting postures during the approximately half-hour duration of playing. The only significant RIP difference in the sitting postures related to less abdominal expansion in the inclined forward seating position, which presumably simply reflects the slightly increased longitudinal dimension of the abdomen in this posture.

The preference for the standing posture can logically be associated with the changes in expansion and muscle activity. The changes in abdominal and chest expansion likely led to feelings of increased breathing control while playing. The decreased magnitude and reduced range of abdominal expansion may be related both to an increase in the superior/inferior dimensions of the abdominal cavity in standing, and also to the increased abdominal muscle activation. It is possible that the musicians experienced some sense of this increased abdominal muscle activity, leading to a feeling of more “breath support” while playing. This is an important component of wind musician performance and is commonly advocated by expert musicians (e.g., Farkas, 1962).

The lack of variation in abdominal muscle activity in the different sitting postures was an unexpected finding in light of previous research suggesting that slouching postures would reduce muscle activity compared to more upright or forward postures (e.g., O'Sullivan et al., 2002; Kera and Maruyama, 2005; Lin et al., 2006; Lee et al., 2010). However, previous studies did not involve musicians playing orchestral repertoire, which involves a wide variety of respiratory demands compared to standard lung function testing parameters. Musicians have been reported to use a range of lung volume (~30%, similar to that observed here) that has in the past been measured as wind musicians initiated breaths at 55–87% of their vital capacity (VC) and terminated breaths at 14–52% VC (Fuks and Sandberg, 1999). In contrast, most other studies measuring lung function in different sitting postures have used measures relating to full vital capacity and tidal volume of the lungs, and without external resistance applied to the outbreath (e.g., O'Sullivan et al., 2002; Kera and Maruyama, 2005; Lin et al., 2006; Lee et al., 2010). Whilst musical repertoire is one reason for differing air volume requirements, different instruments all provide varying levels of resistance to the expired air (Cugell, 1986), requiring more support from the expiratory muscles than seen in quiet breathing (Aliverti, 2008).

In the muscle activity recorded in this study, the standing posture nearly doubled the activation of the upper and lower abdominal muscles in comparison to all seated postures. The higher average value of around 12% MVC of both abdominal muscles in standing compared to about 8% MVC in sitting postures remains acceptable in light of longstanding recommendations of maintaining intermittent repetitive workloads below 14% of MVC for extended durations (Bjorksten and Jonsson, 1977). This information adds to the currently limited EMG research on skill-based analyses of performance in musicians, which has been suggested to be an important way forward in performance and teaching optimization as well as injury prevention (Visentin and Shan, 2011). For example, playing while standing will clearly utilize higher levels of abdominal muscle activity than in sitting, so that orchestral performers who play or practice only in sitting might gradually reduce abdominal strength, which is required when speed or volume demands increase. However, playing seated and using lower levels of abdominal activation may also increase the duration for which a performer can utilize these muscles. More research needs to be conducted to confirm or refute such hypotheses extrapolated from the data presented here.

Despite documented differences in instrument resistance to the expired air (Bouhuys, 1964), in the current study maximum pressure was not a significant covariant in any of the variables. In a previous study comparing maximal expired air pressure in a range of musicians, differences also were not seen between instrument groups (Schorr-Lesnick et al., 1985), with differences observed in only one other study between trumpet players and non-musicians (Fiz et al., 1993). These limited findings point to the need for further research to clarify relative pressure generation mechanisms in different wind musicians. There are several possible reasons for the lack of variability of abdominal activation levels found in the sitting postures used in this study. The expiratory demands of the orchestral excerpts chosen may have not highlighted extreme differences in abdominal activation patterns, or the amount of laryngeal resistance or glottis closure (Farkas, 1962) may not have varied enough.

Musicians will encounter many different seating arrangements in their training and career and they need to maintain their performance quality regardless of this factor. It is possible that honing their playing skill despite variations in available chairs has trained more consistent abdominal muscle activation patterns in a range of sitting postures (“breath support”). It is interesting that the decreased abdominal expansion seen in forward inclined sitting was accompanied by only a marginal increase in abdominal activation that did not reach statistical significance in this study.

The increased muscle activation of the abdomen and the subjective sensation of increased breath support and control in the standing posture, suggests an important use of thoracic respiratory musculature and movements during playing. However, the lack of variability of thoracic expansion movements seen in all postures may reflect that only subtle changes occurred in spinal position and consequently did not largely impact on movements in the thoracic cage as noted in previous research (Lee et al., 2010). Better understanding the usage characteristics of the primary and secondary respiratory muscles acting on the thoracic cage during playing is an important direction for future research. In addition, measures of very specific musical demands, such as vibrato, may result in different activation patterns to other requirements. However, measuring the activation patterns of all different musical performance demands for wind and brass instrumentalists was beyond the scope of the current study.

A possible limitation of this study was the lack of measurement of variations in performance quality in the different playing postures. This was felt to be inappropriate given the musicians' limited practice of the excerpts used in the study, as recordings may have simply improved with repetition. However, sound was recorded using an external microphone to synchronize EMG readings with the performance and this appeared to motivate performers to play as well as possible. Further studies would benefit from including this measure as an indicator of outcomes of altered respiratory mechanics. In addition, the duration of playing over approximately half an hour may not have been adequate to highlight potential changes in muscle activation levels over time related to the sitting posture adopted. Over a longer duration of testing, fatigue patterns may have emerged differently in the various seated postures. However, the duration of the testing in the current study did approximate the duration of a typical practice session.

In summary, woodwind and brass musicians have enhanced abdominal muscle activation and chest expansion characteristics in standing compared to sitting. In sitting, other factors such as player comfort and instrument support may be more important considerations for the individual musician, as one sitting posture cannot be strongly recommended over another on the basis of respiratory mechanics, although sitting reclined was clearly favored least by the musicians.

### Conflict of interest statement

The authors declare that the research was conducted in the absence of any commercial or financial relationships that could be construed as a potential conflict of interest.
